# Endoscopic findings of cardiac lymphoid hyperplasia and *Helicobacter pylori* infection status

**DOI:** 10.1002/deo2.15

**Published:** 2021-08-22

**Authors:** Kyoichi Adachi, Norihisa Ishimura, Takumi Notsu, Kanako Kishi, Tomoko Mishiro, Kazunari Sota, Nahoko Nagano, Shunji Ishihara

**Affiliations:** ^1^ Health Center Shimane Environment and Health Public Corporation Shimane Japan; ^2^ Second Department of Internal Medicine Shimane University Faculty of Medicine Shimane Japan; ^3^ Pathology Unit Shimane University Hospital Shimane Japan

**Keywords:** Barrett's esophagus, blue laser imaging, endoscopy, *Helicobacter pylori*, lymphoid hyperplasia

## Abstract

**Objective:**

Lymphoid hyperplasia is endoscopically observed as multiple small whitish round nodules or spots. This retrospective study was performed to examine the prevalence of that finding in patients with Barrett's epithelium and its relationship with the status of *H. pylori* infection.

**Methods:**

The study subjects were 3353 patients (males 2186, females 1167; mean age 55.2 ± 9.4 years) in whom the status of *H. pylori* infection had been determined. The presence of Barrett's epithelium ≥ 5 mm in length was endoscopically determined, and then endoscopic observations with blue laser imaging were used to investigate the presence of lymphoid hyperplasia in those areas.

**Results:**

Barrett's epithelium was diagnosed in 1884 (56.2%) of the subjects, and endoscopic findings of lymphoid hyperplasia were observed in 402 (21.3%) of those with Barrett's epithelium. Lymphoid hyperplasia in Barrett's epithelium was not present in any without a current or prior *H. pylori* infection, while the prevalence of lymphoid hyperplasia in *H. pylori*‐positive and post‐eradicated subjects was 48.4% and 30.4%, respectively (*p* < 0.001). Multiple logistic regression analysis revealed that female gender, younger age, and higher degree of gastric mucosal atrophy were significant factors related to lymphoid hyperplasia positivity. In addition, the duration after *H. pylori* eradication was negatively correlated with its prevalence.

**Conclusion:**

Endoscopic findings of cardiac lymphoid hyperplasia were well correlated with *H. pylori* infection, although prevalence decreased over time following bacterial eradication.

## INTRODUCTION

**FIGURE 1:**
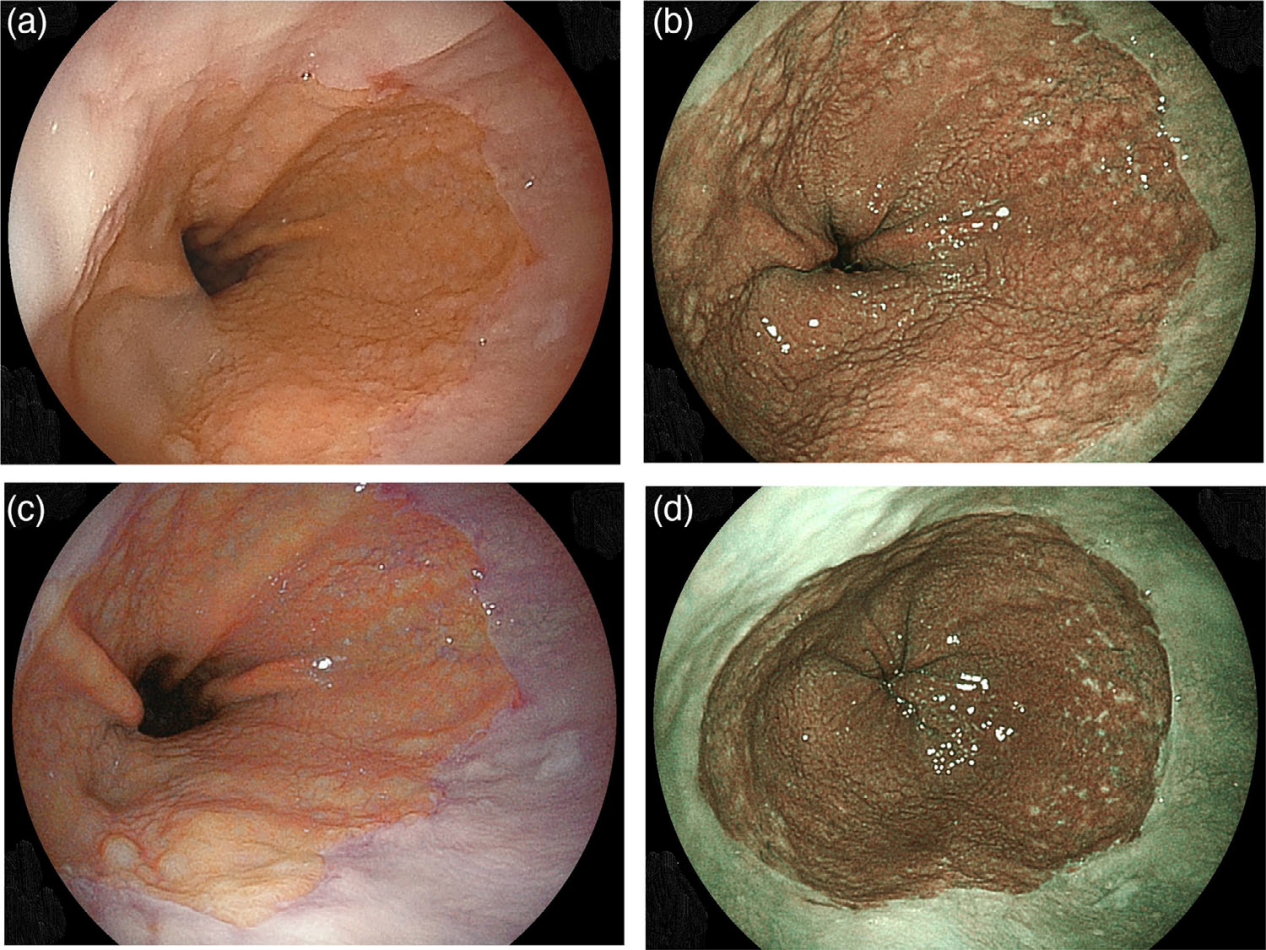
Endoscopic findings of cardiac portion in subjects positive for *H. pylori* infection ((a) white light image, (b) blue laser imaging [BLI], (c) linked color imaging [LCI], (d) BLI image 2 years after eradication for *H. pylori*]. Multiple small whitish round nodules, an endoscopic finding indicating lymphoid hyperplasia, were easily recognized with BLI. In addition, the distal ends of palisade vessels were observed in the area of lymphoid hyperplasia as well as on the oral side of gastric folds with endoscopy using LCI, suggesting that endoscopic findings showing lymphoid hyperplasia can be confirmed in an area of Barrett's epithelium. Endoscopic findings of lymphoid hyperplasia has been diminished after successful eradication for *H. pylori*

**FIGURE 2:**
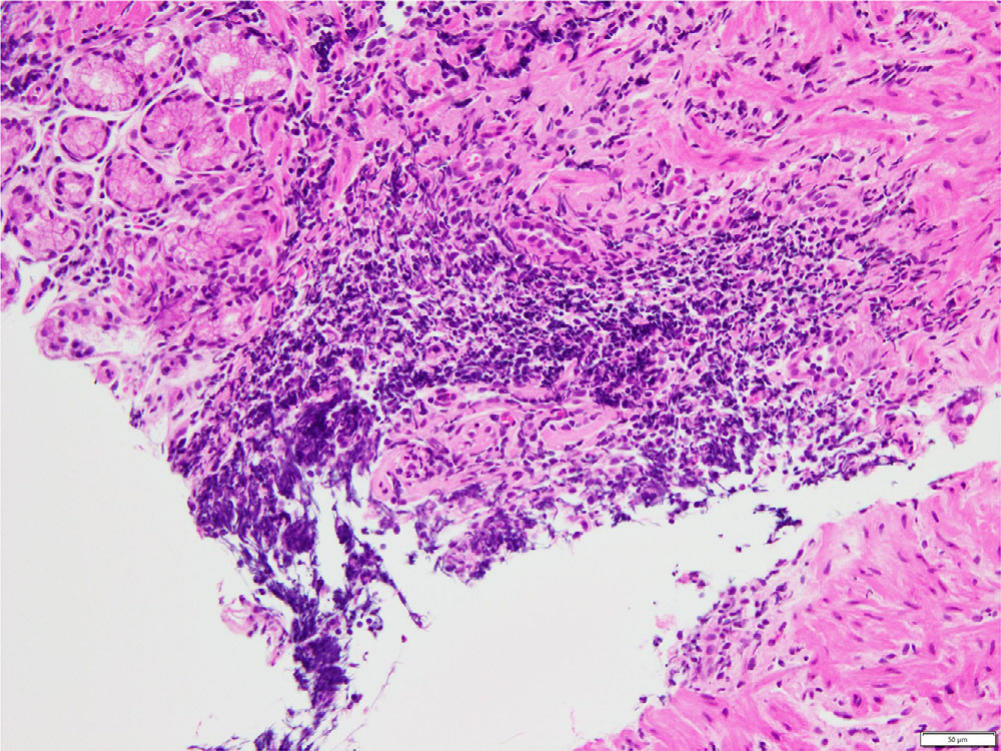
Histology results of biopsied samples obtained from cardiac portion in patients with multiple small whitish round nodules shown by endoscopy. Lymphocyte aggregation was observed deep in the area of mucosa

Endoscopic findings of multiple small whitish round nodules or spots, which morphologically resemble those of nodular gastritis, are sometimes observed in the cardiac portion. Recently, Yamasaki and coworkers reported such endoscopic findings as cardiac gastritis in association with lymphoid hyperplasia.^1^ Such evidence indicating lymphoid hyperplasia is easily recognized with use of narrow‐band imaging or blue laser imaging (BLI) (Figure 1), while histology results of a biopsied sample obtained from a related site show aggregation of lymphocytes (Figure 2).^1^

Based on criteria presented by The Japan Esophageal Society, the esophagogastric junction (EGJ) is defined as the distal margin of the palisade vessels of the lower esophagus, while columnar‐appearing mucosa between the squamocolumnar and the EGJ is diagnosed as Barrett's epithelium.^2^, ^3^ As shown in Figure 1, palisade vessel distal ends can be observed in an area of lymphoid hyperplasia located on the oral side of gastric folds. Therefore, lymphoid hyperplasia formed in Barrett's epithelium can be shown by endoscopy.

To the best of our knowledge, the prevalence and clinical significance of lymphoid hyperplasia in the cardiac portion have yet to be determined. The present study was conducted to examine the prevalence of endoscopic findings of lymphoid hyperplasia in Barrett's epithelium and its relationship with *Helicobacter pylori (H. pylori)* infection, since gastric lymphoid hyperplasia, which is observed in cases of nodular gastritis, is known to be associated with *H. pylori* infection.^4^, ^5^, ^6^

## MATERIALS AND METHODS

### Subjects

The study subjects were individuals who visited the Health Center of Shimane Environment and Health Public Corporation for a detailed medical checkup examination between April 2019 and March 2020, the majority of whom were socially active and productive and considered to be socioeconomically middle class. During the study period, a total of 5061 individuals underwent an upper GI endoscopic examination. Those with a history of gastric surgery or whose status of *H. pylori* infection could not be determined based on medical records and/or serum antibody test findings were excluded from enrollment. Serum anti‐*H. pylori* IgG antibody detection was performed with a SphereLight *H. pylori* antibody J kit, and the antibody titer was automatically determined using a chemiluminescent enzyme immunoassay method, with a value ≥ 4.0 U/ml defined as positive, according to the manufacturer's instructions.^7^, ^8^, ^9^ In addition, a precise medical history concerning the status of *H. pylori* infection (negative, positive, post‐eradication) was obtained in an interview with the subject conducted by a public health nurse. Usage of a proton pump inhibitor or H2 receptor antagonist (H2RA) within the preceding 3 months was also determined at the time of the interview, although details related to duration or dosage of anti‐secretory drugs were not obtained for this study.

Subjects who had undergone therapy but without successful eradication were included in the group with *H. pylori* infection. When eradication therapy was confirmed to be not successful, we recommended that the subject undergo an *H. pylori* stool antigen test at our institution. The presence or absence of *H. pylori* infection and results of eradication therapy were also confirmed based on endoscopic findings obtained in an upper GI endoscopic examination,^10^, ^11^, ^12^, ^13^ which was performed for all of the subjects.

**FIGURE 3:**
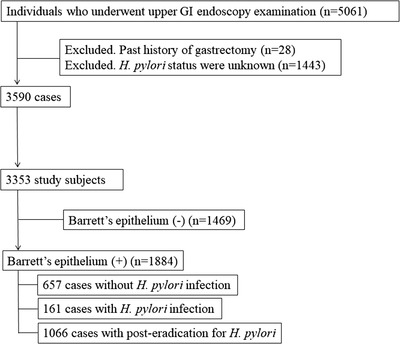
Flow of subject selection for this study

Individuals with endoscopic findings indicating gastric mucosal atrophy classified as C2‐O3 in the classification of Kimura and Takemoto ^14^ and negative in anti‐*H. pylori* IgG antibody test findings were excluded if they had not been treated for eradication of *H. pylori*, since such cases might have been considered as post‐eradication status even though they did not previously undergo eradication therapy or were currently positive for *H. pylori* infection.^8^, ^15^ As a result, the present study cohort was composed of 3353 subjects (males 2186, females 1167; mean age 55.2 ± 9.4 years) in whom the status of *H. pylori* infection was determined (Figure 3).

### Endoscopic findings

All upper GI endoscopic examinations were performed by experienced licensed endoscopists using an EG‐L580NW endoscope (Fujifilm, Tokyo, Japan). At our institution, an upper GI endoscopic examination is performed with the subject in an unsedated condition without anti‐cholinergic drug administration and the endoscope typically inserted in a transnasal manner. The EGJ was endoscopically investigated with the subject asked to inspire deeply. Endoscopic findings using linked color imaging (LCI) were used to determine the area of Barrett's epithelium, as the distal end of the palisade vessels could be easily visualized.^3^, ^16^ For this study, Barrett's epithelium was diagnosed as positive when an area ≥ 5 mm in length was noted halfway around the lower esophagus, as shorter length Barrett's epithelium was observed in nearly all of the cases that underwent endoscopy with LCI. In cases with Barrett's epithelium, endoscopic observations using BLI were performed to determine the presence of lymphoid hyperplasia. In this study, endoscopic finding of lymphoid hyperplasia is defined as positive when multiple small whitish round nodules or spots were observed by BLI observation even if the area was small (Figure 1). Endoscopic observations using BLI to determine the presence of lymphoid hyperplasia were started at our institution in September 2018, and the observation of cardiac portion using BLI had been easily executed by all examiners in this study period (from April 2019 to March 2020).

Endoscopic findings of reflux esophagitis were evaluated using the Los Angeles classification,^17^ and individuals with a grade of A, B, C, or D were diagnosed as positive for reflux esophagitis. Gastric mucosal atrophy, evaluated based on endoscopic findings using the classification of Kimura and Takemoto, was classified into six groups (C1, C2, C3, O1, O2, O3),^14^ which has been shown to correlate well with histological features of atrophy.^18^ For the present study, C1‐C2 was defined as mild, C3‐O1 as moderate, and O2‐O3 as severe gastric mucosal atrophy. The presence of nodular gastritis was also endoscopically determined in each subject.

All endoscopic images from each subject were simultaneously reviewed by three expert endoscopists, and the presence of lymphoid hyperplasia was determined by consensus. In addition, endoscopic findings indicating positive for *H. pylori* infection, such as nodular gastritis, spotty and/or diffuse redness of fundic gland mucosa, and sticky mucous,^10^, ^11^, ^12^, ^13^ were carefully determined in all investigated cases. The prevalence of endoscopic findings of lymphoid hyperplasia was retrospectively analyzed after dividing the cases into groups based on *H. pylori* infection status. In addition, the relationship between lymphoid hyperplasia and duration after eradication in subjects with *H. pylori* post‐eradicated status was examined.

### Statistical analysis

Statistical analyses were performed using a chi‐square test, Mann‐Whitney's U test, and multiple logistic regression analysis. All calculations were performed with the StatView 5.0 software program for Macintosh (Abacus Concepts Inc., Berkeley, CA, USA), and a *p* level < 0.05 was considered to indicate statistical significance.

### Ethics

This study was performed in accordance with the Declaration of Helsinki, and the protocol was approved by the ethics committee of the Shimane Environment and Health Public Corporation (IRB No: 2017‐03). Written informed consent indicating that obtained clinical data would be used for a clinical study without release of individual information was received from all subjects before performing the medical checkup examinations.

## RESULTS

**FIGURE 4:**
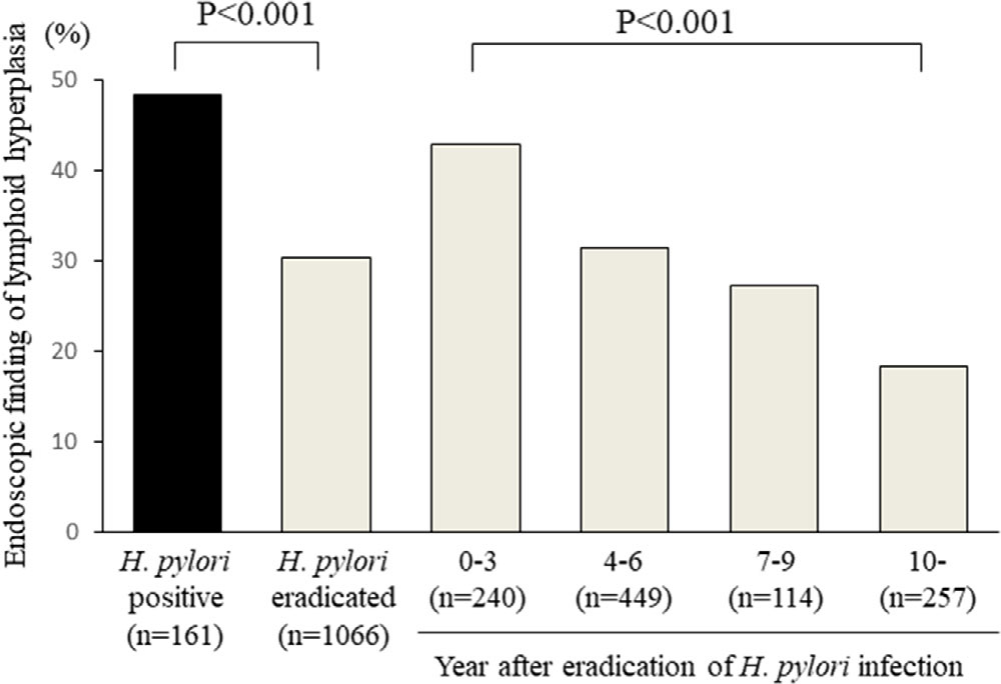
*H. pylori*‐positive and post‐eradicated subjects with endoscopic findings of lymphoid hyperplasia in Barrett's epithelium. The timing of *H. pylori* eradication could not be clarified in six subjects, and those were excluded from the analysis of duration after *H. pylori* eradication

Among the 3353 subjects in the present study, Barrett's epithelium was endoscopically determined in 1884 (56.2%), with findings indicating lymphoid hyperplasia noted in 402 (21.3%) of those. The characteristics of subjects with and without lymphoid hyperplasia in Barrett's epithelium are shown in Table 1. Female predominance and younger age were noted in subjects with lymphoid hyperplasia, while prevalence in *H. pylori*‐positive and post‐eradicated subjects was 48.4% and 30.4%, respectively (*p* < 0.001) (Table 1, Figure 4). In all subjects negative for *H. pylori* infection, no endoscopic evidence of lymphoid hyperplasia was noted. The degree of gastric mucosal atrophy was greater in those with as compared to without lymphoid hyperplasia findings. Nodular gastritis was seen in only eight with lymphoid hyperplasia, although its prevalence was significantly different as compared to subjects without lymphoid hyperplasia. Reflux esophagitis was less frequently observed in subjects with endoscopic finding of lymphoid hyperplasia, and the number of cases with anti‐secretory therapy was smaller in those cases (Table 1). Multiple logistic regression analysis showed that female gender, younger age, and greater degree of gastric mucosal atrophy were significant factors for positive lymphoid hyperplasia findings. A comparison of post‐eradicated subjects with those positive for *H. pylori* infection showed an odds ratio of 0.612 (Table 2).

**TABLE 1:** Characteristics of subjects with endoscopic findings of lymphoid hyperplasia in Barrett's epithelium

	Lymphoid hyperplasia		
	Positive (*n* = 402)	Negative (*n* = 1482)	*p* value
Gender, male/female	251/151	1058/424	<0.001
Age, years	53.7 ± 9.0	56.0 ± 9.2	<0.001
*H. pylori* infection			<0.001
Negative	0	657 (44.3%)	
Positive	78 (19.4%)	83 (5.6%)	
Post‐eradication	324 (80.6%)	742 (50.1%)	
Gastric mucosal atrophy			<0.001
Mild	184 (45.8%)	1091 (73.6%)	
Moderate	181 (45.0%)	305 (20.6%)	
Severe	37 (9.2%)	86 (5.8%)	
Nodular gastritis	8 (2.0%)	6 (0.4%)	0.001
Reflux esophagitis	40 (10.0%)	310 (20.9%)	0.005
Anti‐secretory therapy	15 (3.7%)	114 (7.7%)	<0.001
PPI/H2RA usage	11/4	104/10	

Data are expressed as the mean ± SD or number of subjects. Gastric mucosal atrophy was evaluated using the classification of Kimura and Takemoto (C1‐C2: mild, C3‐O1: moderate, O2‐O3: severe). Reflux esophagitis: Grade A‐D of the Los Angeles classification.

Abbreviations: H2RA, H2 receptor antagonist; PPI, proton pump inhibitor.

**TABLE 2:** Multiple logistic regression analysis of subjects with endoscopic findings of lymphoid hyperplasia in Barrett's epithelium classified as positive for *H. pylori* infection or post‐eradicated status

	Odds ratio	95% CI	*p* value
Gender (female)	1.585	1.217–2.063	<0.001
Age	0.946	0.932–0.960	<0.001
Gastric mucosal atrophy (vs. mild atrophy)			
Moderate	1.600	1.224–2.090	<0.001
Severe	1.648	1.045–2.599	0.032
*H. pylori* infection (vs. positive)			
Post‐eradication	0.612	0.429–0.872	0.007

Subjects without *H. pylori* infection were excluded from analysis, as no endoscopic findings of lymphoid hyperplasia were observed in those. Gastric mucosal atrophy was evaluated using the classification of Kimura and Takemoto (C1‐C2: mild, C3‐O1: moderate, O2‐O3: severe).

When analysis of the relationship of endoscopic findings showing lymphoid hyperplasia and duration after eradication was performed for the *H. pylori*‐eradicated cases, prevalence declined in association with longer duration after eradication (Figure 4). Multiple logistic regression analysis of *H. pylori*‐post‐eradicated cases showed that female gender and younger age were significant factors for positive lymphoid hyperplasia findings, while the duration after *H. pylori* eradication was negatively correlated with prevalence of lymphoid hyperplasia (Table 3).

**TABLE 3:** Multiple logistic regression analysis of subjects with endoscopic findings of lymphoid hyperplasia in Barrett's epithelium classified as post‐eradicated status following *H. pylori* infection

	Odds ratio	95% CI	*p* value
Gender (female)	1.571	1.177–2.098	0.002
Age	0.955	0.939–0.971	<0.001
Gastric mucosal atrophy (vs. mild atrophy)			
Moderate	1.309	0.975–1.758	0.073
Severe	1.443	0.877–2.375	0.149
Duration after eradication (years)	0.933	0.904–0.963	<0.001

Six subjects for whom the timing of *H. pylori* eradication could not be clarified were excluded from analysis. Gastric mucosal atrophy was evaluated using the classification of Kimura and Takemoto (C1‐C2: mild, C3‐O1: moderate, O2‐O3: severe).

## DISCUSSION

In this study, we examined the prevalence of endoscopic findings of lymphoid hyperplasia in Barrett's epithelium and the relationship with *H. pylori* infection status. That was observed in 402 (21.3%) of the 1884 cases with Barrett's epithelium and clearly demonstrated to be associated with *H. pylori* infection. None of the subjects classified as without *H. pylori* infection had endoscopic findings indicating lymphoid hyperplasia. Furthermore, its prevalence was significantly higher in *H. pylori*‐positive subjects as compared to those classified as post‐eradicated (48.4% vs. 30.4%).

Endoscopic findings of lymphoid hyperplasia in Barrett's epithelium resemble those of nodular gastritis, which are characteristically observed in cases positive for *H. pylori* infection.^4^, ^5^, ^6^ Histology results of nodular gastritis shows that it consists of lymphoid follicles, which is also noted in endoscopic findings of lymphoid hyperplasia in the cardiac portion.^1^ Female predominance and younger age were observed in the present subjects with lymphoid hyperplasia in Barrett's epithelium, characteristics also commonly reported in cases of nodular gastritis.^4^, ^18^, ^19^, ^20^ Therefore, it is considered that the same mechanism is related to both conditions, although the mechanism of lymphoid hyperplasia occurrence has not been clarified. Endoscopic findings of nodular gastritis are mainly observed in younger patients with mild gastric mucosal atrophy, including pediatric cases, and considered to develop during an early stage of *H. pylori* infection.^4^, ^19^, ^20^, ^21^ On the other hand, a higher degree of gastric mucosal atrophy was observed in the present subjects with lymphoid hyperplasia in Barrett's epithelium, although only a few showed nodular gastritis. Therefore, we speculated that lymphoid hyperplasia in Barrett's epithelium might be formed at a later stage of bacterial infection, while the same mechanism may have an important role for formation of not only nodular gastritis but also lymphoid hyperplasia in affected patients. Development of gastric mucosal atrophy has been repeatedly demonstrated to correlate with low prevalence of gastroesophageal reflux disease and reduced gastric acid secretion.^18^, ^22^, ^23^, ^24^ Indeed, the results of this study showed that endoscopic finding of lymphoid hyperplasia in Barrett's epithelium was inversely correlated with the presence of reflux esophagitis and the usage of anti‐secretory drugs.

The prevalence of endoscopic findings of lymphoid hyperplasia in Barrett's epithelium gradually declined as the duration after eradication of *H. pylori* was extended in the present cohort. In addition, duration following *H. pylori* eradication was negatively correlated with prevalence of lymphoid hyperplasia. These findings indicated that lymphoid hyperplasia in Barrett's epithelium may be formed during a period of *H. pylori* positivity and then gradually disappears following its successful eradication, as shown in Figure 1. In addition, flat type of endoscopic findings of lymphoid hyperplasia is considered to form after successful eradication for *H. pylori*, as it is also commonly observed after eradication for *H. pylori* in cases with nodular gastritis.

The results of this study demonstrated that the presence of endoscopic finding of lymphoid hyperplasia in Barrett's epithelium should be recognized as an indicator of a present *H. pylori* infection in the stomach or post‐eradicated status. In addition, lymphoid hyperplasia in cardiac portion may have some important carcinogenic function, since nodular gastritis has been shown to have a correlation with gastric cancer incidence.^19^, ^20^ The influence of cardiac lymphoid hyperplasia on future occurrence of cancer in cardiac portion should be investigated in long‐term prospective study, and the significance of disappearance of it after eradication for *H. pylori* is needed to be clarified.

The present study has several limitations, including the enrolled cohort, which was comprised of socially active and productive individuals who visited a medical center for a medical check‐up, with the number of younger subjects relatively few. The prevalence of nodular gastritis has been demonstrated to be high in younger ages,^4^, ^19^, ^20^, ^21^ thus additional studies are needed to determine the relationship between presence of nodular gastritis and lymphoid hyperplasia in younger subjects. All endoscopic images were simultaneously reviewed by three expert endoscopists to determine the presence of endoscopic findings of lymphoid hyperplasia. Therefore, inter‐observer differences regarding diagnosis could not be examined. In addition, we did not examine the histological finding of cardiac lymphoid hyperplasia in almost all cases, since the study subjects were individuals who visited for a detailed medical checkup examination. Thus, the association between endoscopic findings and histological grade of lymphoid hyperplasia could not be determined. Furthermore, this study was performed in a cross‐sectional manner at a single medical center. A prospective multi‐center study is recommended for determining the effects of continuous *H. pylori* infection as well as its eradication on time‐course endoscopic and histological changes of cardiac lymphoid hyperplasia.

In conclusion, endoscopic findings of cardiac lymphoid hyperplasia were noted in 56.2% of 1884 subjects with Barrett's epithelium. Those findings were well correlated with *H. pylori* infection and prevalence decreased over time following its eradication.

## CONFLICT OF INTEREST

The authors have no conflict of interest to declare in regard to this study.

## FUNDING INFORMATION

None.
